# Optimized breeding strategies for multiple trait integration: I. Minimizing linkage drag in single event introgression

**DOI:** 10.1007/s11032-013-9936-7

**Published:** 2013-08-15

**Authors:** Ting Peng, Xiaochun Sun, Rita H. Mumm

**Affiliations:** 1 Department of Crop Sciences and the Illinois Plant Breeding Center, University of Illinois at Urbana-Champaign, 1102 S. Goodwin Ave., Urbana, IL 61801 USA; 2 Monsanto Company/Seminis Vegetable Seeds, Felda, FL USA; 3 Dow AgroSciences, Indianapolis, IN USA

**Keywords:** Computer simulation, Multiple trait integration, Marker-aided backcross, Single event introgression, Breeding strategy, Linkage drag

## Abstract

From a breeding standpoint, multiple trait integration (MTI) is a four-step process of converting an elite variety/hybrid for value-added traits (e.g. transgenic events) using backcross breeding, ultimately regaining the performance attributes of the target hybrid along with reliable expression of the value-added traits. In the light of the overarching goal of recovering equivalent performance in the finished conversion, this study focuses on the first step of MTI, single event introgression, exploring the feasibility of marker-aided backcross conversion of a target maize hybrid for 15 transgenic events, incorporating eight events into the female hybrid parent and seven into the male parent. Single event introgression is conducted in parallel streams to convert the recurrent parent (RP) for individual events, with the primary objective of minimizing residual non-recurrent parent (NRP) germplasm, especially in the chromosomal proximity to the event (i.e. linkage drag). In keeping with a defined lower limit of 96.66 % overall RP germplasm recovery (i.e. ≤120 cM NRP germplasm given a genome size of 1,788 cM), a breeding goal for each of the 15 single event conversions was developed: <8 cM of residual NRP germplasm across the genome with ~1 cM in the 20 cM region flanking the event. Using computer simulation, we aimed to identify optimal breeding strategies for single event introgression to achieve this breeding goal, measuring efficiency in terms of number of backcross generations required, marker data points needed, and total population size across generations. Various selection schemes classified as three-stage, modified two-stage, and combined selection conducted from BC1 through BC3, BC4, or BC5 were compared. The breeding goal was achieved with a selection scheme involving five generations of marker-aided backcrossing, with BC1 through BC3 selected for the event of interest and minimal linkage drag at population size of 600, and BC4 and BC5 selected for the event of interest and recovery of the RP germplasm across the genome at population size of 400, with selection intensity of 0.01 for all generations. In addition, strategies for choice of donor parent to facilitate conversion efficiency and quality were evaluated. Two essential criteria for choosing an optimal donor parent for a given RP were established: introgression history showing reduction of linkage drag to ~1 cM in the 20 cM region flanking the event and genetic similarity between the RP and potential donor parents. Computer simulation demonstrated that single event conversions with <8 cM residual NRP germplasm can be accomplished by BC5 with no genetic similarity, by BC4 with 30 % genetic similarity, and by BC3 with 86 % genetic similarity using previously converted RPs as event donors. This study indicates that MTI to produce a ‘quality’ 15-event-stacked hybrid conversion is achievable. Furthermore, it lays the groundwork for a comprehensive approach to MTI by outlining a pathway to produce appropriate starting materials with which to proceed with event pyramiding and trait fixation before version testing.

**Electronic supplementary material:**

The online version of this article (doi:10.1007/s11032-013-9936-7) contains supplementary material, which is available to authorized users.

## Introduction

Since the commercial debut of transgenic crops in the mid-1990s (Koziel et al. 1993; Delannay et al. 1995), the demand for genetically modified (GM) crops has risen dramatically, driven mainly by rapid adoption by US farmers. The adoption rate for GM corn increased from 25 % in 2000 to 88 % in 2012 in the USA (USDA ERS 2012). In addition, there has been a general trend toward GM crops that offer more than one value-added trait per cultivar. For example, historical data provided by USDA indicates that the prevalence of ‘stacked’ trait corn hybrids has increased from 1 % in 2000 to 52 % in 2012 in the US (USDA ERS 2012). Beyond the US, the adoption rates in other countries, especially in some developing countries such as China and Brazil, are also very high, as the benefits of increased farm income (e.g. US$14 billion globally in 2010; US$78.4 billion since 1996) and the decreased environmental impact associated with pesticide usage and greenhouse gas emission from agriculture (e.g. adoption of GM crops led to the equivalent of removing 8.6 million cars from the road in 2010) are quite attractive (Brookes and Barfoot 2012).

The array of value-added traits has been expanding and now, between those commercialized and those in development, includes herbicide tolerances, insect resistances, drought tolerance, nitrogen use efficiency, yield enhancement, grain composition modification (e.g. amino acid composition, protein content, and oil profile), disease resistances, grain processing (e.g. phytase for animal feed and amylase for corn ethanol), and others (Information Systems for Biotechnology 2012). Furthermore, for traits which may elicit a resistance response such as resistance by targeted insect pests, the trend has been to combine multiple modes of action to stave off development of resistance (Que et al. 2010). This trend toward stacking of more and more value-added traits is expected to continue and even escalate. By 2030, it is predicted that as many as 15–20 value-added traits may be offered in new corn hybrids (Que et al. 2010; Fraley 2012).

With such great benefits both economically and environmentally, transgenic trait options will be an important component in crop genetic improvement to close the yield gap. To feed the global population of nine billion people using essentially the same amount of land and less water, the scientific community has committed to doubling or even tripling various crop yields in the next few decades. GM traits will be a key component for achieving this goal, along with conventional breeding practices, advanced breeding technologies [e.g. QTL (quantitative trait loci)) mapping, genomic-assisted selection], and improved agricultural practices e.g. increased plant density, optimized tillage practices (Moose and Mumm 2008; Monsanto Company 2012).

Nowadays, marker technology is widely used as an aid in introgressing target genes or transgenic events [an event is defined as the unique DNA sequence inserted in the host genome through transformation and the precise point of insertion (Mumm and Walters 2001)] into a target hybrid or, more specifically, the recurrent parent (RP) lines used to produce the hybrid. In this study, with maize as a model crop, we evaluate breeding strategies for integrating up to 15 transgenic events in a given hybrid via computer simulation. Although we have focused on transgenic events, the results could be easily extended to other types of target genes including major QTL (Ribaut and Ragot 2007) and genes from exotic sources (Young and Tanksley 1989; Bernardo 2009). The overall objective of multiple trait integration (MTI) is to integrate the specific transgenic events conferring the value-added trait phenotypes into the elite genetic package represented by the target hybrid, regaining the performance attributes of the target hybrid along with reliable expression of the value-added traits. Typically, molecular markers are utilized in MTI for efficiency, speed, and improved probability of recovering equivalent performance in the converted hybrid relative to the unconverted target hybrid.

**Fig. 1:**
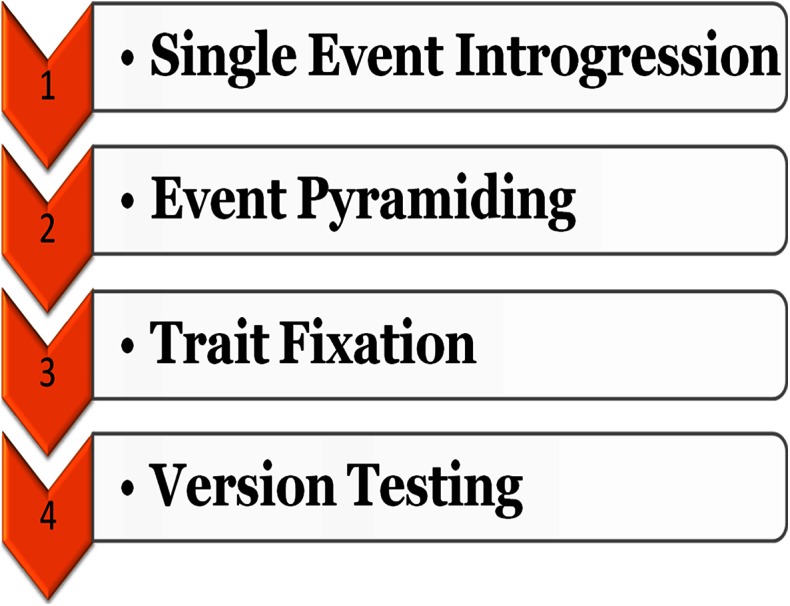
Four steps involved in multiple trait integration (MTI) in maize: single event introgression, event pyramiding, trait fixation, and version testing (performance testing of various versions of a given target hybrid conversion)

The MTI process in maize is comprised of four essential steps: single event introgression, event pyramiding, trait fixation, and version testing (performance testing of various versions of a given target hybrid conversion; Fig. 1). For single event introgression, the breeding goal is to introgress a single event from a donor parent into the RP through backcross breeding, achieving a high rate of recovery of RP germplasm. With MTI, single event introgression streams for a target RP are designed to be conducted in parallel. The goal for event pyramiding is to assemble all the specified events in the target RP by crossing single-event conversions. All event loci are in heterozygous state at the close of the first two steps. The goal for trait fixation is to recover at least one line which is homozygous for all event loci to ensure stable expression of value-added traits. In order to minimize the risk of failure in recovering the target hybrid performance, typically multiple versions of the RP conversions are generated and yield-tested (Mumm and Walters 2001). Conversions of the parent lines are hybridized to produce various versions of the converted target hybrid, which are then evaluated as to performance relative to the unconverted target hybrid. The main goal for version testing is to ensure that all the characteristics of the target hybrid have been recovered in at least one version of the converted target hybrid.

Success of MTI is achieved with the recovery of at least one version of the converted target hybrid with equivalent performance to the unconverted target hybrid and stable expression of all the value-added traits. Thus, a ‘quality’ conversion is necessary. Without achieving this outcome, all upstream efforts and resource investments are of no or limited value. The probability of success depends largely on the amount of non-recurrent parent (NRP) germplasm that can be eliminated in the MTI process. Several studies (Stam and Zeven 1981; Young and Tanksley 1989; Hospital 2001) have shown that the majority of the residual NRP germplasm in a given RP conversion is closely linked to the target gene (or event) being introgressed, a phenomenon known as linkage drag and attributable to the low probability of double recombinants very near the target locus. For this reason, a number of studies (e.g. Ishii and Yonezawa 2007; Frisch and Melchinger 2001) have emphasized the need for single event introgression prior to stacking. Our simulation study confirmed this finding. Computer simulation demonstrated that the effectiveness of linkage drag elimination is much less when donors carrying multiple events are utilized, holding effective population size and number of generations of breeding equal (data not shown).

With MTI, the issue of linkage drag is magnified in proportion to the number of events being introgressed. The residual NRP germplasm may contain deleterious genes, genes associated with negative interactions, or germplasm composition from a different heterotic group that may impact expression of heterosis in the converted hybrid. There are three potential scenarios that can affect the ability to achieve this goal: use of a non-elite transformation line, e.g. Hi-II derived from A188 and B73 (Armstrong et al. 1991); somaclonal variation resulting from tissue culture during the transformation process; and use of a donor parent from the opposite heterotic group (e.g. donor from the female heterotic group to convert a line from the male heterotic group). Nowadays, elite transformation lines are often used in biotech trait development, making the latter situation especially pertinent to conversions produced with newly developed events. Since all events originate from a single plant (T0 plant regenerated from a transformed cell), if some traits are to be introgressed into the opposite heterotic group, there is a greater risk of failure to recover a RP conversion with equivalent performance due to potential reduction of heterosis.

One approach is to designate an upper bound for the amount of residual NRP germplasm in the converted target hybrid consistent with a high probability of recovering equivalent yield performance. Furthermore, when stacking events, a stricter threshold of RP germplasm recovery relative to single trait conversion is required to have a high likelihood of recovering equivalent performance in a multi-trait cultivar. This translates to high stringency applied in single event introgression in MTI for each individual conversion. For example, if a threshold of ≥96.66 % RP germplasm recovery (i.e. ~120 cM of NRP germplasm in maize; see Eq. 5) is required to achieve equivalent performance in the converted hybrid, the outcome of single event introgression conducted in parallel streams must achieve ≤8 cM NRP germplasm, which is consistent with 99.78 % RP germplasm recovery in individual event conversions, to stack 15 events. Under such a strict selection criterion, reduction of linkage drag becomes the hurdle to fully recover the RP germplasm and, ultimately, performance of the target hybrid.

Introgressing as many as 15 events is complex as there are numerous ways to achieve this breeding goal. In addition to a high probability of success in recovering a converted target hybrid with equivalent performance to its unconverted counterpart, other considerations, namely time to market and resource allocation, must be considered in choosing a breeding strategy for MTI. The breeding strategy, therefore, must address parameters including desired outcomes for each generation, selection scheme, number of backcross generations, number of marker data points required, population size, and selection intensity in each generation. The breeding strategy for each step must be optimized in keeping with immediate priorities and goals, yet integrated throughout the entire process of MTI for a successful comprehensive approach.

Numerous studies have been conducted to optimize breeding strategies for marker-aided backcross breeding, with the aim of reducing the number of generations required, minimizing total population size, and minimizing the number of marker data points [see reviews by Visscher et al. (1996), Ribaut et al. (2002), Frisch (2005)]. Ribaut et al. (2002) concluded that to achieve more than 99 % of RP germplasm recovery, marker-aided selection must be applied to all backcross generations. Others highlighted the value of applying marker-aided selection in later backcross generations rather than earlier (Hospital et al. 1992; Frisch et al. 1999; Ribaut et al. 2002). Some studies have recommended selection against linkage drag in early backcross generations to take advantage of the relatively larger amount of genetic variation (Frisch et al. 1999; Herzog and Frisch 2011). Frisch (2005) proposed various selection schemes for use in marker-aided backcross breeding, mainly two-stage selection, three-stage selection, and four-stage selection. Two-stage selection consists of selection for the target gene or event, followed by selection for RP germplasm recovery (background selection). Three-stage selection strategy consists of one step of target gene selection; one step of selection against linkage drag in the chromosomal region immediately adjacent to the target gene facilitated by two markers flanking the gene (or event), and a last step of RP germplasm recovery selection by markers across the genome. Four-stage selection dissects the background selection in the three-stage selection into two steps: RP germplasm recovery selection on the carrier chromosome (chromosome with the target gene) and RP germplasm recovery selection on non-carrier chromosomes (all chromosomes in the genome except the carrier chromosome). Frisch et al. (1999) recommended using a three-stage selection or four-stage selection method to reduce the linkage drag. Likewise, Falke et al. (2009) concluded that a three-stage selection method is the most efficient in reducing linkage drag.

Most studies to date have not considered MTI overall and none to date have considered the scenario involving introgression of 15 events. Furthermore, previous studies may not have taken into account the availability of very dense marker coverage of the genome, allowing for deployment of strategies that might not otherwise be possible, such as intense selection in the chromosomal region flanking the event insertion by dense markers. In addition, there has been little work published to assess choice of donor parent in trait integration. Yet, within a seed company, there may be numerous options available to a breeder in choosing a donor for a particular event, particularly as time from market launch of the event increases.

The objectives of this study were twofold: first, to identify optimal breeding strategies for MTI using computer simulation, focusing on efficiencies for single event introgression to achieve successful conversion of a target hybrid for 15 events. Criteria for evaluating efficiency include amount of total residual NRP germplasm in the finished conversion (Total NRP), amount of NRP germplasm remaining in the chromosomal region flanking the event insertion site (FR NRP), total number of marker data points (MDP), total number of plants grown across generations (NPG), and total number of generations. Note that optimization of other steps in the MTI process is addressed in other publications (see Peng et al. 2013; Sun 2012) included in a series that centers on a comprehensive approach to MTI of 15 events, as we see this as a realistic objective for the not-too-distant future in plant breeding. Secondly, we have evaluated strategies for choice of donor parent to facilitate conversion efficiency and quality based on introgression history and genetic similarity between the donor parent and the RP. Criteria for evaluating efficiencies relate to time and resource investment.

We developed a realistic breeding scenario that might be encountered in the seed industry which assumes that (1) the transformation line is considered to be related to the female side of the heterotic pattern, (2) some events are required on the male side of the target hybrid; therefore, to balance out the number of events for introgression into each parent, eight events are to be introgressed into the female RP and seven events into the male RP; (3) all events are new so conversions for each event are required; (4) events for conversion of a given RP are not linked genetically (i.e. each event is located on different chromosome); (5) FR NRP will be virtually unalterable after the single event introgression step is completed and event pyramiding begins; (6) 120 cM of NRP germplasm (~96.66 % RP recovery) is the upper limit of residual NRP germplasm consistent with a high probability of recapturing target hybrid performance (Sun 2012, Chapter 4). With 15 events overall, this requires ≤8 cM residual NRP germplasm in each single event conversion. Furthermore, because we assumed that FR NRP will be nearly impossible to alter after single event introgression is completed and event pyramiding begins, we arbitrarily designated the threshold for FR NRP for each single event introgression to be ~1 cM.

We used computer simulation to investigate the various breeding strategies for single event introgression in MTI and for choice of donor parent. Computer simulation can be useful in efficiency studies as it enables comparison of numerous approaches which can ultimately lead to improvements in the breeding process, enabling greater speed to market, rate of gain, resource savings, and innovative outcomes (Sun et al. 2011).

## Materials and methods

### Genetic simulation

Computer simulations in this study were conducted using R statistical software (v2.10.1); models of the genome and the MTI advancement process were developed. The genome model for simulation was constructed according to the published maize ISU–IBM genetic map, with a total length of 1,788 cM (Fu et al. 2006). Genetic recombination was simulated by Haldane’s mapping function (Haldane 1919; Prigge et al. 2008) and the random walk algorithm (Crosby 1973), assuming no crossover interference. Genetic markers were evenly spaced across the chromosomes every 1 cM, for a total of 1,798 markers across the genome (two end markers were simulated at the ends of each chromosome).

To facilitate selection for each event, a single marker serving as a perfect marker for the event was utilized. To select against linkage drag, ten markers spaced 1 cM apart on each side of an event locus were utilized. This 20 cM region was considered the event flanking region. To track NRP germplasm in selection for RP germplasm recovery, markers distributed uniformly at 20 cM intervals were utilized. For selection schemes involving more than one element of selection in a given generation, event selection (ES), selection against linkage drag in the 20 cM region flanking the transgenic event (LDS), and selection for the recurrent parent germplasm recovery (RPS) were conducted in tandem.

Individual plant scores for LDS were calculated according to Eqs. 1 and 2 below. The linkage score for an individual (*S_LD*) is calculated as the summation across all marker loci *i* through *n* of the product of the weight for each LDS marker in the flanking region (*W_LD*) and individual marker genotypic score (*G_LD*) (Eq. 2). The weight of each LDS marker is calculated by the portion of adjusted distance (*D*_*i*_) (10 cM minus the absolute distance from the marker to the event loci) to the total adjusted distance to the event position at each side of the event loci (Eq. 1).

1 $$ W\_LD_{i} = \frac{{10 - D_{i} }}{{\sum\nolimits_{i = 1}^{n} {(10 - D_{i} )} }} $$

2 $$ S\_LD = \sum\limits_{i = 1}^{n} {W\_LD_{i} *G\_LD} $$

In this way, all the marker weights for one side of the flanking region around the event can be summed to 1 as a way of standardizing the LDS scores for each genotype being screened, and occurrences of recombination which happen near to the event can be given more weight than occurrences of recombination which happen relatively far from the event. A similar calculation was used by Hospital et al. (1992). The genotypic score for each individual LDS marker is counted as 1 if the LDS marker locus is homozygous (1, 1), or 0 if the LDS marker loci is heterozygous (0, 1). The simulated backcross progeny were ranked according to the calculated LDS scores, and then, in accordance with the selection intensity, a certain number of individuals with highest LDS scores were selected.

Individual plant scores for RPS (*S_RP*) were calculated as the summation across all marker loci *i* through *n* of the product of the weight (*W_RP*) and the genotypic score (*G_RP*) for each RPS marker (Eq. 4). The weight is calculated by the average coverage of the total genome based on the mean of the distances [left marker interval distance *D*(*l*)_*i*_ and right marker interval distance *D*(*r*)_*i*_ to the two adjacent markers] (Eq. 3). As with the LDS score calculation, the genotypic value for each RPS markers is counted as 1 if the RPS marker locus is homozygous (1, 1) and 0 if the RPS marker locus is heterozygous (0, 1).

3 $$ W\_RP_{i} = \frac{{D(l)_{i} - D(r)_{i} }}{2} $$

4 $$ S\_RP = \sum\limits_{i = 1}^{n} {W\_RP_{i} *G\_RP} $$

Various levels of genetic similarity can be simulated by adjusting the number of polymorphic markers and monomorphic markers in the full set. In the study of optimized breeding strategies for single event introgression, 100 % polymorphic markers were used for simulation and calculation. For choice of donor parent with different genetic similarity level with recurrent parent, different percentages of polymorphic markers were simulated. Marker values were set as outlined below for the donor parent and the recurrent parent at each locus. In order to track event presence among the backcross progeny, the event marker value is set to 1 in the donor parent and 0 in the recurrent parent. To track the recurrent parent germplasm recovery through backcross generations, if the marker is polymorphic, then the donor parent marker value is 0 and the recurrent parent marker value is 1. If the marker is monomorphic, then both the donor parent marker and recurrent parent marker values are 1. Thus, in the final backcrossing stages, the desired genotype would be homozygous (1, 1) for every marker locus except the event marker locus which would be heterozygous (1, 0).

The process model was used to create progeny genotypes produced through crossing, backcrossing, or self-pollination and accounts for the results of selection in each generation. The default for population size was 400 progeny with selection of four individuals as parents for the next generation in generations involving LDS or RPS, and a population size of eight was simulated if applying event selection only. To evaluate the effect of population size on efficiency in single event introgression, population sizes of 20, 50, 100, 200, 400, 600, 800, 1,000, 1,500, and 2,000 were considered. To evaluate the effect of selection intensity on efficiency in single event introgression, the number of selected individuals was varied. For each simulation, the mean of 1,000 repeats was used in order to minimize random error.

### Developing a reference population

Before the comparison of breeding strategies, a reference population was created to serve as a baseline for relative efficacy. We simulated ten generations of backcrossing with 1,000 individuals per generation, with selection for only the event of interest, and computed the mean and the standard deviation of the residual NRP germplasm across the whole genome, the carrier chromosome (chromosome with the event), the non-carrier chromosomes (chromosomes other than the one with event), and a 20 cM flanking region around the event. Furthermore, in order to observe the effectiveness of the RPS on linkage drag elimination, we simulated ten generations of backcrossing with 1,000 individuals in each generation, applying event selection plus recurrent parent (ES + RPS) selection. Likewise, event selection plus selection against linkage drag (ES + LDS) was applied for ten backcross generations with 1,000 individuals per generation.

### Comparison of selection schemes

Single event introgression was simulated using a number of different selection schemes, including three-stage selection, modified two-stage selection, and combined selection methods. Three-stage selection features tandem selection, first for event presence, then for favorable recombinants in the flanking region around the event, and lastly for RP germplasm recovery across the entire genome (ES + LDS + RPS), all in the same backcross generation (following Frisch 2005). However, while Frisch (2005) chose all the favorable recombinants in the flanking region and selected one ‘best’ individual with the greatest RP germplasm recovery, our approach is to select a certain number of ‘best’ lines based on LDS scores and, out of those lines, select a certain number of ‘best’ lines based on RPS scores. To facilitate comparisons among selection schemes, all involve selection of the top 2 % for LDS scores, from which the best 50 % of individuals for RPS scores are selected. We implemented a modified two-stage selection with selection for event presence followed by either selection for RP germplasm recovery (ES + RPS) or linkage drag selection (ES + LDS). The combined selection method involves the combination of the modified two-stage method including linkage drag selection (ES + LDS) and the three-stage selection method (ES + LDS + RPS) across various generations of selection. Various selection schemes for three to five backcross generations of the marker-aided backcross breeding program with constant population size of 400 were evaluated. We also evaluated outcomes achieved with specific selection schemes, varying population size from 20 to 2,000.

Criteria considered in comparing efficiencies among selection schemes included: total amount of residual NRP germplasm in the total genome (Total NRP), amount of residual NRP in the flanking region (FR NRP), total number of marker data points required (MDP), and total number of plants grown (NPG) and number of generations required. Ribaut et al. (2002) defined the efficiency indicator for each marker-aided backcross breeding program as the ratio between the resources that need to be invested at each generation and the number of generations required in order to achieve the selection goal. Other simulation studies (e.g. Frisch et al. 1999) defined the percentage of the RP germplasm recovered across the genome (RP%) in selected genotypes as the efficiency indicator. Here, we utilized a similar efficiency indicator; however, we measured residual non-recurrent parent germplasm and expressed this statistic as a length in cM rather than a percentage of RP recovery. This addressed our concerns about the accumulation of NRP germplasm, particularly that which originates from donor parents on the opposite side of the heterotic pattern, in integrating multiple events into one maize hybrid. The relationship between RP% and Total NRP can be expressed as follows:

5 $$ {\text{Total}}\;{\text{NRP}}\left( {\text{cM}} \right) = \left( {1 - {\text{RP}}\% } \right)*{\text{Genome length}}*2 $$

where genome length is denoted in cM.

Note that with this formula each strand of DNA in a diploid individual is not considered independently in accounting for NRP germplasm; thus, chromosomal segments heterozygous for NRP germplasm are considered to be NRP overall, which has particular importance with non-homozygous individuals.

In addition, when we compared results from different selection schemes, we considered NRP in the flanking region (FR NRP) as the first comparison criterion and NRP in the total genome (Total NRP) as the second comparison criterion. The reason is that large NRP in the total genome can be easily reduced by one or more additional generations of backcrossing even without marker-aided selection whereas large NRP in the flanking region is harder to reduce, requiring large population sizes and marker-aided selection, i.e. more resource expenditure. Finally, we also estimated the total marker data points (MDP) required and total population size (individual plants grown; NPG) for each breeding strategy in order to facilitate comparison of the total resource requirement for each breeding strategy. For each breeding scheme, Total NRP, FR NRP, MDP, and NPG were computed in simulations based on 1,000 repeats.

### Choice of donor parent

Introgression history of the target event donor and genetic similarity between donor parent and RP were the two main factors evaluated for their impact on choice of donor parent. FR NRP (cM) in each backcross generation was recorded to observe the linkage drag in the flanking region. A number of levels of genetic similarity between the donor parent and the RP, from low to high, were simulated: genetic similarity = 0.00, 0.10, 0.20, 0.30, 0.40, 0.50, 0.60, 0.70, 0.80, 0.83, 0.86, 0.89, 0.90, 0.92, 0.95, and 0.98. With all simulations of genetic similarity, genetic differences were randomly distributed across the genome.

## Results and discussion

### Reference population baseline

Before the comparison of selection schemes, a reference population composed of 1,000 individuals was created to serve as a baseline for relative efficacy in evaluating breeding strategies. According to quantitative genetic theory, the residual NRP germplasm decreases by half with each successive backcross generation while the proportion of recovered RP germplasm increases in step. Considering a genetic map of 1,788 cM length in total [e.g. the maize map as per Fu et al. (2006)], the mean amount of NRP germplasm (in cM) can be related to the percentage of RP germplasm recovered in each generation according to Eq. 5 (see Supplemental Table S1). Thus, Total NRP is expressed more conservatively than percentage of RP germplasm, as it considers marker loci for which the RP conversion is in heterozygous state as unconverted loci rather than half converted. For example, in the BC1 generation, the mean percentage of RP germplasm recovered is 75 % whereas a mean total of 899 cM of the genome still contains residual NRP germplasm.

Applying selection for only the event to be introgressed (i.e. ES) from BC1 through BC10 in the reference population (population size = 1,000 in each generation, repeats = 1,000), the mean Total NRP is higher than the amount expected without selection (Table S1, Table 1a). Furthermore, comparing the amount of NRP germplasm on the carrier chromosome (chromosome with the event insertion), non-carrier chromosomes (all other chromosomes except the one with event), and 20 cM flanking region around the event, the carrier chromosome has a disproportionate amount of residual NRP germplasm (Table 1a). Moreover, the rate at which the NRP decreases with backcrossing was much slower for the carrier chromosome, particularly for the chromosomal region flanking the event insertion site (Table 1a). Clearly, computer simulation shows that selection for the event only, either by perfect marker or by phenotype, is ineffective in reducing linkage drag as suggested in earlier studies (e.g. Young and Tanksley 1989). However, marker-aided selection in the flanking region should be helpful in targeting and eliminating linkage drag.

**Table 1:** Baseline results (population size = 1,000 per generation, repeats = 1,000) for selection during single event introgression based on event selection (ES), linkage drag selection in the 20 cM region flanking the transgenic event (LDS), recurrent parent germplasm selection (RPS) or a combination across ten generations. (a) With ES only, the mean of the NRP and standard deviation in the total genome (Total NRP), on the carrier chromosome (CC NRP), on the non-carrier chromosomes (NC NRP) and in the flanking region around the event (FR NRP) from BC1 to BC10. (b) With ES + RPS, the mean of the NRP in the total genome (Total NRP) and in the flanking region around the event (FR NRP) from BC1 to BC10. (c) With ES + LDS, the mean of the NRP in the total genome (Total NRP) and in the flanking region around the event (FR NRP) from BC1 to BC10

Generation	Total NRP (cM)		CC NRP (cM)		NC NRP (cM)		FR NRP (cM)	
	Mean	SD	Mean	SD	Mean	SD	Mean	SD
(a)								
BC1	1,398.79	431.38	158.17	40.69	1,240.62	403.62	19.53	1.77
BC2	973.9	453.62	127.01	49.18	846.89	423.2	18.88	2.65
BC3	681.45	403.5	103.32	49.11	578.13	374.36	18.26	3.22
BC4	480.31	337.1	85.16	46	395.15	310.68	17.66	3.65
BC5	343.2	274.41	71.71	42.05	271.49	250.95	17.12	3.96
BC6	248.19	220.79	60.96	37.99	187.23	199.98	16.58	4.21
BC7	182.57	177.39	52.45	34.21	130.12	158.86	16.08	4.42
BC8	135.83	141.39	45.63	30.7	90.2	125	15.58	4.6
BC9	103.09	112.95	40.16	27.64	62.93	98.38	15.11	4.74
BC10	79.43	90.4	35.66	24.94	43.77	77.4	14.65	4.85

Generation	BC1	BC2	BC3	BC4	BC5	BC6	BC7	BC8	BC9	BC10
(b)										
Total NRP (cM)	452.83	100.44	21.09	14.93	14.29	13.75	13.31	12.89	12.5	12.07
FR NRP (cM)	17.99	15.59	12.54	11.97	11.73	11.55	11.36	11.17	10.96	10.72
(c)										
Total NRP (cM)	903.18	424.45	312.84	236.3	182.44	140.31	108.29	84.23	66.04	51.74
FR NRP (cM)	9.65	1.84	1.08	0.99	0.96	0.95	0.94	0.93	0.92	0.91

Applying selection for the event and the RP germplasm recovery in tandem (ES + RPS) from BC1 through BC10 in the reference population (population size = 1,000 in each generation, repeats = 1,000), the effectiveness in reducing the Total NRP is shown (Table 1b). However, this selection scheme was not effective in reducing the NRP germplasm in the flanking region (Table 1b). A large amount of residual NRP germplasm remained in the flanking region even at BC10; that is, of the total residual NRP of 12.07 cM in the genome, 10.72 cM was situated in the flanking region. Again it is apparent that marker-aided selection in the flanking region is necessary to effectively address linkage drag, particularly if there is to be any possibility of achieving the defined breeding goal of ~1 cM NRP in the flanking region.

Applying selection for the event and against the linkage drag in tandem (ES + LDS) from BC1 through BC10 in the reference population (population size = 1,000 at each generation, repeats = 1,000), it was shown that if dense markers in the flanking region around the event (e.g. one per cM in the 20 cM region) are used to facilitate the elimination of linkage drag, the linkage drag can be decreased to approximately 1 cM by BC4 (Table 1c). However, it is also apparent that the linkage drag is difficult to reduce further even after many more backcrosses to the RP; at BC10, 0.91 cM of NRP remains in the flanking region on average. Thus, it is clearly possible to reduce linkage drag to 1 cM in the region flanking the event insertion with marker-aided selection but difficult to reduce it much beyond 1 cM due to the low chance of recombination in this limited chromosomal region. Considering the results in Table 1 as baselines and examples of lower bounds in response to selection against NRP germplasm, a balance between selection for ES, RPS, and LDS will be crucial for successfully converting a maize hybrid for 15 events at a given germplasm recovery rate.

### Breeding strategy comparison

#### Optimal selection scheme

For simulation, we considered selection schemes classified as three-stage, modified two-stage, and combined selection conducted from BC1 through BC3, BC4, or BC5 with constant population size (8/400 individuals) and selection intensity (four individuals) at each generation. With three-stage selection, selection for ES, LDS, and RPS was conducted in tandem in the same generation. With modified two-stage selection, either LDS or RPS was selected within a generation after ES selection, but not both. With combined selection, one type of scheme or the other might be conducted within a generation (Table 2; also Tables S2, S3). Typically, LDS is conducted prior to RPS to take advantage of greater genetic variation in earlier BC generations and/or in the first step of tandem selection (Young and Tanksley 1989; Frisch et al. 1999; Ribaut et al. 2002). Three generations of marker-aided backcross selection have been espoused for adequate recovery of the RP genome (Ribaut et al. 2002). However, given the very stringent breeding goal to recover a RP conversion with <8 cM NRP and ~1 cM NRP in the flanking region at the close of single event introgression, it is apparent that three generations of selection were not sufficient with any selection scheme (Table S2). Among all nine proposed breeding schemes implemented through BC3, Total NRP across breeding schemes ranged from 244.12 to 28.75 cM (equivalent to 93.21–99.20 % RP recovery) and FR NRP across breeding schemes ranged from 10.78 to 2.02 cM. Minimal Total NRP (28.75 cM) was obtained using the modified two-stage breeding scheme ES + LDS/ES + RPS/ES + RPS from BC1 to BC3 whereas minimal FR NRP (2.02 cM) was obtained with the combined breeding scheme ES + LDS/ES + LDS/ES + LDS + RPS from BC1 to BC3 (Table S2). However, none of the three-generation breeding schemes met the defined breeding target.

**Table 2:** Comparison of breeding strategies for single event introgression under constant population size of 400 and four selected genotypes per generation for five backcross generations based on selection for event selection (ES), linkage drag selection in the 20 cM region flanking the transgenic event (LDS), and recurrent parent selection (RPS), displaying the mean of total non-recurrent parent germplasm length in cM (Total NRP), the flanking region non-recurrent parent germplasm length in cM (FR NRP), the genotyped marker data points in thousands (MDP), and total population size (NPG) (1,000 repeats)

Selection schemes	BC1	BC2	BC3	BC4	BC5	Total NRP (cM)	FR NRP (cM)	MDP (1,000)	NPG
Three-stage	ES + LDS + RPS	ES + LDS + RPS	ES + LDS + RPS	ES + LDS + RPS	ES + LDS + RPS	17.85	1.42	222.000	2,000
	ES	ES + LDS + RPS	ES + LDS + RPS	ES + LDS + RPS	ES + LDS + RPS	31.02	1.8	177.608	1,608
	ES	ES	ES + LDS + RPS	ES + LDS + RPS	ES + LDS + RPS	47.7	2.67	133.216	1,216
	ES	ES	ES	ES + LDS + RPS	ES + LDS + RPS	75.68	5.17	88.824	824
	ES	ES	ES	ES	ES + LDS + RPS	148.28	9.73	44.432	432
Modified two-stage	ES + LDS	ES + RPS	ES + RPS	ES + RPS	ES + RPS	14.83	7.81	166.000	2,000
	ES + LDS	ES + LDS	ES + RPS	ES + RPS	ES + RPS	8.65	3.43	130.000	2,000
	*ES + LDS	ES + LDS	ES + LDS	ES + RPS	ES + RPS	7.86	1.68	94.000	2,000
	ES + LDS	ES + LDS	ES + LDS	ES + LDS	ES + RPS	19.17	1.27	58.000	2,000
	ES	ES + LDS	ES + RPS	ES + RPS	ES + RPS	14.68	7.5	125.608	1,608
	ES	ES + LDS	ES + LDS	ES + RPS	ES + RPS	9.59	3.09	89.608	1,608
	ES	ES + LDS	ES + LDS	ES + LDS	ES + RPS	21.86	1.69	53.608	1,608
	ES	ES	ES + LDS	ES + RPS	ES + RPS	16.38	7.54	85.216	1,216
	ES	ES	ES + LDS	ES + LDS	ES + RPS	21.47	3.13	49.216	1,216
	ES	ES	ES	ES + LDS	ES + RPS	39.27	7.94	44.824	824
Combined	ES + LDS	ES + LDS + RPS	ES + LDS + RPS	ES + LDS + RPS	ES + LDS + RPS	21.16	1.33	182.000	2,000
	ES + LDS	ES + LDS	ES + LDS + RPS	ES + LDS + RPS	ES + LDS + RPS	30.99	1.26	142.000	2,000
	ES + LDS	ES + LDS	ES + LDS	ES + LDS + RPS	ES + LDS + RPS	54.87	1.19	102.000	2,000
	ES + LDS	ES + LDS	ES + LDS	ES + LDS	ES + LDS + RPS	108.24	1.15	62.000	2,000
	ES	ES + LDS	ES + LDS + RPS	ES + LDS + RPS	ES + LDS + RPS	40.53	1.61	137.608	1,608
	ES	ES + LDS	ES + LDS	ES + LDS + RPS	ES + LDS + RPS	64.04	1.49	97.608	1,608
	ES	ES + LDS	ES + LDS	ES + LDS	ES + LDS + RPS	117.18	1.38	57.608	1,608
	ES	ES	ES + LDS	ES + LDS + RPS	ES + LDS + RPS	69.41	2.25	93.216	1,216
	ES	ES	ES + LDS	ES + LDS	ES + LDS + RPS	117.05	1.94	53.216	1,216
	ES	ES	ES	ES + LDS	ES + LDS + RPS	113.32	4.63	48.824	824

The breeding strategy marked with * shows potential to meet the defined breeding goal for <8 cM Total NRP and FR NRP ~1 cM

Considering selection schemes involving selection through BC4, 16 breeding schemes were evaluated (Table S3). The mean Total NRP across breeding schemes ranged from 210.74 to 10.62 cM (equivalent to 94.14–99.70 % RP recovery) and FR NRP across breeding schemes ranged from 10.49 to 1.45 cM. Minimal Total NRP (10.62 cM) was obtained with the modified two-stage breeding scheme ES + LDS/ES + LDS/ES + RPS/ES + RPS from BC1 to BC4 whereas the minimal FR NRP (1.45 cM) was obtained with the combined breeding scheme ES + LDS/ES + LDS/ES + LDS/ES + LDS + RPS from BC1 to BC4. Again, with up to four generations of marker-aided selection conducted with a constant population size of 400, the specified breeding target was not realized.

Considering selection schemes involving selection for five backcross generations (Table 2), 25 breeding schemes were evaluated for Total NRP, FR NRP, MDP and NPG. As shown in the table, Total NRP ranged from 148.28 to 7.86 cM (equivalent to 95.88–99.78 % RP recovery) and FR NRP ranged from 9.73 to 1.15 cM. One selection scheme met the breeding goal of <8 cM Total NRP (marked with an asterisk in Table 2). This selection scheme involved three generations of selection for ES + LDS followed by two generations of selection for ES + RPS. Nonetheless, with this scheme, the FR NRP was estimated at 1.68 cM. Although there were several selection schemes that met the breeding goal of ~1 cM FR NRP (the minimal FR NRP was 1.15 cM), none of these was adequate to reduce the Total NRP to <8 cM.

Even though the difference between 1 and 1.68 cM seems small, if we convert centiMorgans into base pairs of DNA sequence, the small differential would represent thousands of base pairs. Thus, we considered two ways to improve this situation: (1) adding one more backcross generation, or (2) increasing the population size and/or selection intensity. By adding one more backcross generation of event and linkage drag selection, the breeding goal for FR NRP could certainly be met (data not shown). However, sometimes adding one more backcross generation can lead to a year’s delay in commercial release of the new value-added product. Thus, we also pursued the second option, evaluating the impact of increased population size and selection intensity. We simulated the ‘best’ BC5 selection scheme using larger population sizes in the generations from BC1 to BC3 (population size = 600, 800, and 1,000 per generation) when LDS is under selection, and holding population sizes in the BC4 and BC5 generations at 400 when the rest of the genome is under selection (since the Total NRP breeding goal had been met). With increasing the BC1–BC3 population size to 600, the FR NRP decreased from 1.68 cM (with population size 400) to 1.18 cM (Table S4). If greater reduction of FR NRP is desired, population sizes can be inflated to 1,000 individuals during BC1–BC3 to provide reduction of FR NRP to 1.07 cM. Thus, we concluded that with the modified two-stage selection scheme ES + LDS/ES + LDS/ES + LDS/ES + RPS/ES + RPS from BC1 to BC5 and population sizes of 600 and above for BC1–BC3 and 400 for BC4–BC5, the breeding goal of <8 cM Total NRP and ~1 cM FR NRP for single event introgression with each of 15 events could be achieved. To meet the defined breeding goal in this manner, resource requirements would be increased modestly; MDPs were increased by 6,600 and the total population size (NPG) was increased from 2,000 to 2,600 with populations of 600 in BC1 through BC3.

#### Impact of population size and selection intensity

**Fig. 2:**
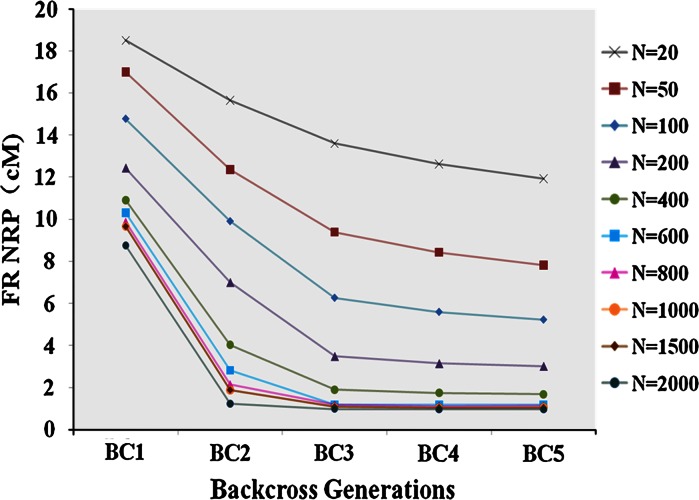
Impact of population size (population size = 20, 50, 100, 200, 400, 600, 800, 1,000, 1,500, 2,000) per generation on the rate of decrease in amount of linkage drag (FR NRP) with selection scheme ES + LDS/ES + LDS/ES + LDS/ES + RPS/ES + RPS from BC1 to BC5 with constant selection intensity 0.01

In general, as population size increases through the backcross process, the Total NRP and the FR NRP decrease more rapidly. Given the selection scheme ES + LDS/ES + LDS/ES + LDS/ES + RPS/ES + RPS with 0.01 selection intensity each generation, the Total NRP target is reached at BC5 with a population size of 400 (Table 2). However, holding population size constant across backcross generations, the FR NRP target of ~1 cM is not achieved at BC5 with population size of 400 (1.68 cM), is achieved at BC3 with population size of 600 (1.20 cM) and 800 (1.15 cM), and is approached at BC2 with population size 2,000 (1.23 cM; Fig. 2). Thus, increasing population size could help the breeder to accelerate the conversion process and save time to market in release of new value-added cultivars, especially when linkage drag elimination is a defined breeding target. Designing the appropriate breeding strategy is a choice between resource and time saving. By balancing the resource requirement and time, one can design the optimal breeding plan based on the specific objectives of the actual breeding program.

Likewise, increasing selection intensity by selecting fewer individuals per round is yet another way to hasten recovery of the RP germplasm. We assumed four individuals chosen from each generation of selection, which is reasonable in real-life scenarios yet rigorous. Reducing the number of individuals selected to one or two per generation does result in a more rapid decrease in Total NRP and FR NRP, generally speaking, especially with a large population size (data not shown). However, one has to take into consideration the related risk associated with a single individual selection (plant/seed) from which to produce the next generation (e.g. germination failure). In addition, the seed needs to generate the desired population size in the next generation must be taken into account. The kernels obtained from a single ear may not be sufficient to plant the next generation at the planned population size. Thus, multiple selected individuals are suggested, with population size adjusted in keeping with the predesigned proportion of selected individuals desired.

### Choice of donor parent

Up to this point, we have considered only ‘first-wave’ conversions, that is, conversions using the transformant line as the donor, as with new events in the product pipeline. Once first-wave conversions have been completed, these converted RPs represent additional potential choices of donor parents to use in producing ‘second-wave’ conversions. Several years after creation of a new transgenic event that is moving toward commercial release, industry breeders can access numerous options for choice of donor parents for a given event. We hypothesized that the ideal donor parent is one that offers quality in terms of less linkage drag, particularly linkage drag representing germplasm from the opposite heterotic group, and higher efficiency in terms of less breeding time. Computer simulation indicated that it is possible to reduce linkage drag in the 20 cM region flanking the event insertion to ~1 cM and that it is difficult to substantially reduce it further (Table 1c). It therefore seems reasonable to accept ~1 cM FR NRP as a first criterion for an optimal donor. Of course, there is still interest in converting the flanking region to the RP at hand but the risk of failure in recovering performance equivalency, especially for conversions in the heterotic group opposite the transformant line, is minimized with this approach.

Among all potential donor parents with minimized linkage drag, a secondary criterion to consider may be genetic similarity between the target RP and the potential donor. Genetic similarity would anticipate that some chromosomal segments in the potential donor may be identical by descent or at least alike in state to those in the target RP. These similar chromosomal segments are in essence already converted to the RP genotype, essentially speeding recovery of the RP germplasm. The impact of the genetic similarity of the donor depends on the level of genetic relationship with the target RP. By using the optimal breeding strategy above, introgression must go to BC5 to achieve <8 cM Total NRP when the donor is unrelated to the target RP (Table 3a). Using a donor that is ≥80 % genetically similar to the RP, the breeding target can be achieved in BC4 (Table 3a). With an alternative breeding strategy comprising two generations of ES + LDS selection and two generations of ES + RPS selection using the same population size and selection intensity, introgression can be completed by BC4 with a donor that is has little as ≥30 % genetic similarity to the RP, and by BC3 with ≥86 % genetic similarity (Table 3b). Thus, simulation shows that the estimated genetic relationship of the potential donor can be taken into account to guide the choice of selection scheme to enable faster recovery of the RP germplasm. Since many companies routinely fingerprint elite lines that may serve as RPs to collect a genotypic profile of proprietary materials, genetic similarity between a given RP and lines previously converted for an event of interest could be easily calculated and used to guide the choice of donor parent for greater efficiency in MTI.

**Table 3:** The impact of genetic similarity between the target recurrent parent (RP) and donor parent in reducing total residual non-recurrent parent germplasm (Total NRP) in a conversion to <8 cM, given two selection schemes: (a) involving three generations of E + LD selection and two generations of E + RP selection and (b) involving two generations of E + LD selection and two generations of E + RP selection

Generation	Selection scheme	Donor similarity range															
		Low genetic similarity									High genetic similarity						
		0 %	10 %	20 %	30 %	40 %	50 %	60 %	70 %	80 %	83 %	86 %	89 %	90 %	92 %	95 %	98 %
(a)																	
BC1	ES + LDS	885.45	798.46	701.99	624.31	526.21	441.47	347.82	268.35	178.79	154.68	117.27	100.08	87.97	73.42	46.55	15.48
BC2	ES + LDS	442.35	391.38	344.34	310.51	265.98	228.7	175.76	138.18	89.49	75.21	60.29	50.47	44.54	36.01	23.53	11.36
BC3	ES + LDS	290.53	256.42	228.11	195.08	169.56	145.61	116.42	88.43	58.62	50.8	39.76	32.45	30.73	24.44	16.11	8.73
BC4	ES + RPS	29.15	24.32	22.19	18.86	16.25	14.51	11.79	8.87	5.68	5.11	4.1	3.38	3.17	2.49	1.86	1.31
BC5	ES + RPS	7.86	7.38	6.24	5.76	4.98	4.24	2.96	2.33	1.54	1.33	1.19	0.86	0.8	0.58	0.48	0.28
(b)																	
BC1	ES + LDS	882.23	793.19	702.43	624.31	526.21	441.47	347.82	268.35	178.79	154.68	117.27	100.08	87.97	73.42	46.55	15.48
BC2	ES + LDS	440.85	392.58	348.22	310.51	265.98	228.7	175.76	138.18	89.49	75.21	60.29	50.47	44.54	36.01	23.53	11.36
BC3	ES + RPS	54.04	47.64	41.42	38.97	32.2	27.22	20.89	17.24	10.85	9.4	7.96	6.26	5.46	4.26	2.89	1.75
BC4	ES + RPS	10.55	9.39	8.18	7.79	6.57	5.62	4.02	3.31	2.07	1.72	1.59	1.21	1.04	0.76	0.61	0.31

## Conclusions

Following the trend of incorporating more and more value-added traits, especially transgenic traits, into newly developed cultivars, it is not unrealistic to anticipate a breeding program in future integrating up to 15 transgenic events in a single maize hybrid in an effort to protect the genetic potential of the hybrid and fill the yield gap. Furthermore, to meet the defined breeding goal of <120 cM NRP in a converted target hybrid, each of the 15 single event introgressions must meet the standard of <8 cM residual NRP germplasm in total across the genome with only ~1 cM NRP germplasm in the region flanking the event insertion. Exploring various breeding strategies through computer simulation to determine whether this ambitious breeding goal is achievable, we have determined that indeed it is. One breeding selection scheme which comprises three generations of selection for the event and against linkage drag in the 20 cM flanking region around the event with population sizes of ≥600 followed by two generations of selection for event and the recurrent parent germplasm recovery throughout the genome with population sizes of ≥400 brings the desired result in selected BC5 individuals. This is a modified two-stage selection scheme which efficiently achieves the goal with modest resource investment. It represents a good balance between selection for elimination of linkage drag and RP recovery across the genome compared with other selection schemes. Furthermore, it takes advantage of the greater genetic variation in the earlier backcross generations to focus selection against linkage drag which has less probability of success than minimization of NRP germplasm throughout the genome. We further conclude that, with the same number of generations of marker-aided selection, gain from RPS is best implemented in later backcross generations to take advantage of gains from backcross breeding per se. Moreover, compared to three-stage selection schemes, two-stage schemes are generally more efficient because the separation of LDS and RPS by generation allows for higher selection intensity per generation per type of selection with minimal increase in MDP. In addition, the selection scheme required adjustment with appropriate population size and selection intensity to accomplish the breeding goal. The optimal breeding strategy featured populations of at least 600 in BC1 through BC3 and populations of 400 in BC4 and BC5, with six and four selected individuals, respectively, to move forward to the next generation. Although selection of fewer individuals in each generation does show some advantage in speeding recovery of the RP germplasm, there is more risk of failure involved with such intense selection and typically seed needs for the next generation cannot be met. Thus, in our proposed breeding strategy, multiple individuals were selected to create the next breeding generation. This optimized breeding strategy supports the conclusions of Herzog and Frisch (2011), highlighting a larger population size in early generations for linkage drag elimination and a smaller population size in later generations for recurrent parent germplasm recovery.

The results of this study can be used as a direct reference for designing a trait integration breeding program aimed at minimizing the risk associated with linkage drag. Under such general guidance, one can customize the optimal breeding strategy based on available resources and specific breeding goals. While the example in the case study presented here was one of introgressing transgenic events, the optimal breeding strategy would also similarly apply to introgressing other genetic factors such as QTL and endogenous genes. For these, modifications such as the use of flanking markers or haplotypes to track the QTL or gene to be introgressed are envisioned.

By computer simulation, we have also established two criteria for choosing an optimal donor parent for a given RP: introgression history showing reduction of linkage drag to ~1 in the 20 cM region flanking the event insertion and genetic similarity between the RP and potential donor parents. The former minimizes risks associated with failure to recover equivalent performance due to diluting the heterotic pattern or unfavorable genetics from a non-elite transformant line or somaclonal variation arising from the transformation process. The latter facilitates acceleration of the conversion process. Simulation demonstrated that a ‘quality’ single event conversion (<8 cM residual NRP in the case of a 15-event stack in the converted hybrid) can be accomplished by BC5 with no genetic similarity, by BC4 with a donor of 30 % genetic similarity to the RP, and by BC3 with 86 % genetic similarity. In a large-scale trait integration program, especially in the seed industry, with these two criteria appropriate computer software tools could be created that would systematically manage the donor parent pool and direct the choice of a donor parent, resulting a faster introgression with higher quality.

This study focused on the first step in MTI, single event introgression. It lays the groundwork for a comprehensive approach to MTI from single event introgression, to event pyramiding, to trait fixation, and to version testing in order to recover a 15-event conversion of a target hybrid with equivalent performance. The reader is referred to Peng et al. (2013) and Sun (2012, Chapter 4) for simulation results pertaining to the other steps in MTI. Finally, the conclusions of this work offer a direct reference for maize breeding and can also help with formulation of conversion strategies in other crops (either inbred or hybrid) to meet defined breeding goals.

## Electronic supplementary material

Below is the link to the electronic supplementary material.

Supplementary material 1 (DOC 230 kb)

## Abbreviations

CC: Chromosome carrying the event locus
cM: centiMorgan
ES: Event selection
FR NRP: Amount of non-recurrent parent germplasm in the 20 cM region flanking the transgenic event
GM: Genetically modified
LDS: Selection against linkage drag in the 20 cM region flanking the transgenic event
MDP: Total number of marker data points
NC: Chromosomes other than the one carrying an event locus
NRP: Non-recurrent parent
MTI: Multiple trait integration
NPG: Total number of plants grown across generations
QTL: Quantitative trait loci
RP: Recurrent parent
RPS: Selection for the recurrent parent germplasm recovery
Total NRP: Total amount of non-recurrent parent germplasm across the genome
