# Supplementary material for: Optimized breeding strategies for multiple trait integration: I. Minimizing linkage drag in single event introgression
Source: Mol Breed. 2013 Aug 15;33(1):89–104. doi: 10.1007/s11032-013-9936-7 (PMC3890577; doi:10.1007/s11032-013-9936-7)
Supplement: Supplementary file 1 — Supplementary material 1 (DOC 230 kb) [file 11032_2013_9936_MOESM1_ESM.doc]

**Optimized breeding strategies for multiple trait integration: I. Minimizing linkage drag in single event introgression**

Ting Peng, Xiaochun Sun, and Rita H. Mumm*

**Supplementary Information**

**Table S1** In the absence of selection, the theoretical mean percentage of recurrent parent germplasm recovered (RP%) in successive backcross generations versus the mean total amount of non-recurrent parent germplasm (Total NRP) (length in cM) based on formula given below. Note that the genome length = 1788 according to the maize map by Fu et al. (2006).

| **Generation** | **BC1** | **BC2** | **BC3** | **BC4** | **BC5** | **BC6** | **BC7** | **BC8** | **BC9** | **BC10** |
| --- | --- | --- | --- | --- | --- | --- | --- | --- | --- | --- |
| **RP%** | 75.0000 | 87.5000 | 93.7500 | 96.8750 | 98.4375 | 99.2188 | 99.6094 | 99.8047 | 99.9023 | 99.9512 |
| **Total NRP (cM)** | 899.0000 | 449.5000 | 224.7500 | 112.3750 | 56.1875 | 28.0938 | 14.0469 | 7.0234 | 3.5117 | 1.7559 |

*Total*
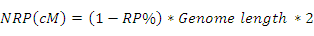


where genome length is denoted in cM

**Table S2** Comparison of breeding strategies for Single Event Introgression under constant population size 400 and 4 selected genotypes per generation for three backcross generations based on selection for event selection (ES), linkage drag selection in the 20 cM region flanking the transgenic event (LDS), recurrent parent selection (RPS), displaying the mean of total non-recurrent parent germplasm length in cM (Total NRP), flanking region non-recurrent parent germplasm length in cM (FR NRP), the genotyped marker data points in thousands (MDP) and total population size (NPG) (1000 repeats).

| **Selection Schemes** | **BC1** | **BC2** | **BC3** | **Total**  **Non-RP(cM)** | **FR**  **Non-RP(cM)** | **MDP**  **(K)** | **NPG** |
| --- | --- | --- | --- | --- | --- | --- | --- |
| **Three-Stage** | ES+LDS+RPS | ES+LDS+RPS | ES+LDS+RPS | 120.65 | 2.91 | 133.2 | 1200 |
|  | ES | ES+LDS+RPS | ES+LDS+RPS | 178.36 | 5.93 | 88.808 | 808 |
|  | ES | ES | ES+LDS+RPS | 299.8 | 10.78 | 44.416 | 416 |
| **Modified**  **Two-Stage** | ES+LDS | ES+RPS | ES+RPS | 28.75 | 8.48 | 85.2 | 1200 |
|  | ES+LDS | ES+LDS | ES+RPS | 51.87 | 3.61 | 49.2 | 1200 |
|  | ES | ES+LDS | ES+RPS | 101.33 | 9.16 | 44.808 | 808 |
| **Combined** | ES+LDS | ES+LDS+RPS | ES+LDS+RPS | 152.03 | 2.49 | 93.2 | 1200 |
|  | ES+LDS | ES+LDS | ES+LDS+RPS | 221.43 | 2.02 | 53.2 | 1200 |
|  | ES | ES+LDS | ES+LDS+RPS | 244.12 | 4.99 | 48.808 | 808 |

**Table S3** Comparison of breeding strategies for Single Event Introgression under constant population size 400 and 4 selected genotypes per generation for four backcross generations based on selection for event selection (ES), linkage drag selection in the 20 cM region flanking the transgenic event (LDS), recurrent parent selection (RPS), displaying the mean of total non-recurrent parent germplasm length in cM (Total NRP), flanking region non-recurrent parent germplasm length in cM (FR NRP), the genotyped marker data points in thousands (MDP) and total population size (NPG) (1000 repeats).

| **Selection Schemes** | **BC1** | **BC2** | **BC3** | **BC4** | **Total**  **NRP(cM)** | **FR**  **NRP(cM)** | **MDP**  **(K)** | **NPG** |
| --- | --- | --- | --- | --- | --- | --- | --- | --- |
| **Three-Stage** | ES+LDS+RPS | ES+LDS+RPS | ES+LDS+RPS | ES+LDS+RPS | 48.94 | 1.88 | 177.6 | 1600 |
|  | ES | ES+LDS+RPS | ES+LDS+RPS | ES+LDS+RPS | 75.76 | 2.87 | 133.208 | 1208 |
|  | ES | ES | ES+LDS+RPS | ES+LDS+RPS | 112.53 | 5.53 | 88.816 | 816 |
|  | ES | ES | ES | ES+LDS+RPS | 210.74 | 10.49 | 44.424 | 424 |
| **Modified Two- Stage** | ES+LDS | ES+RPS | ES+RPS | ES+RPS | 16.35 | 8.03 | 125.6 | 1600 |
|  | ES+LDS | ES+LDS | ES+RPS | ES+RPS | 10.62 | 3.66 | 89.6 | 1600 |
|  | ES+LDS | ES+LDS | ES+LDS | ES+RPS | 29.2 | 1.83 | 53.6 | 1600 |
|  | ES | ES+LDS | ES+RPS | ES+RPS | 20.33 | 8.01 | 85.208 | 1208 |
|  | ES | ES+LDS | ES+LDS | ES+RPS | 33.86 | 3.5 | 49.208 | 1208 |
|  | ES | ES | ES+LDS | ES+RPS | 55.46 | 9.76 | 44.816 | 816 |
| **Combined** | ES+LDS | ES+LDS+RPS | ES+LDS+RPS | ES+LDS+RPS | 59.69 | 1.69 | 137.6 | 1600 |
|  | ES+LDS | ES+LDS | ES+LDS+RPS | ES+LDS+RPS | 85.41 | 1.49 | 97.6 | 1600 |
|  | ES+LDS | ES+LDS | ES+LDS | ES+LDS+RPS | 150.58 | 1.45 | 57.6 | 1600 |
|  | ES | ES+LDS | ES+LDS+RPS | ES+LDS+RPS | 108.03 | 2.38 | 93.208 | 1208 |
|  | ES | ES+LDS | ES+LDS | ES+LDS+RPS | 167.22 | 2.06 | 53.208 | 1208 |
|  | ES | ES | ES+LDS | ES+LDS+RPS | 161.44 | 4.65 | 48.816 | 816 |

**Table S4** The effect of increased population size in BC1 through BC3 on the mean of total non-recurrent parent germplasm length in cM (Total NRP), flanking region non-recurrent parent germplasm length in cM (FR NRP), the genotyped marker data points in thousands (MDP) and total population size (NPG) in achieving the specified breeding goal in Single Event Introgression of < 8 cM Total NRP and ~1 cM FR NRP with constant selection intensity 0.01.

| **Generations** | **BC1** | **BC2** | **BC3** | **BC4** | **BC5** | **Total**  **NRP (cM)** | **FR**  **NRP (cM)** | **MDP**  **(K)** | **NPG** |
| --- | --- | --- | --- | --- | --- | --- | --- | --- | --- |
| **Selection Scheme** | **(ES+LDS)** | **(ES+LDS)** | **(ES+LDS)** | **(ES+RPS)** | **(ES+RPS）** |  |  |  |  |
| **Population**  **Size per generation** | 400 | 400 | 400 | 400 | 400 | 7.86 | 1.68 | 94.000 | 2000 |
|  | 600 | 600 | 600 | 400 | 400 | 6.57 | 1.18 | 100.600 | 2600 |
|  | 800 | 800 | 800 | 400 | 400 | 6.10 | 1.13 | 107.200 | 3200 |
|  | 1000 | 1000 | 1000 | 400 | 400 | 5.96 | 1.07 | 113.800 | 3800 |
